# Case report: Histological findings of peri-appendicitis in three children with SARS-CoV-2 – related multisystem inflammatory syndrome: A mark for systemic inflammation?

**DOI:** 10.3389/fped.2022.975940

**Published:** 2022-11-17

**Authors:** Marianna Fabi, Francesco Vasuri, Fiorentina Guida, Alessandro Rocca, Mario Lima, Antonietta D’Errico, Marcello Lanari

**Affiliations:** ^1^ Division of Pediatric Emergency, IRCCS Azienda Ospedaliero-Universitaria di Bologna, Bologna, Italy; ^2^ Pathology Unit, IRCCS Azienda Ospedaliero-Universitaria di Bologna, Bologna, Italy; ^3^ Speciality School of Pediatrics, Sant'Orsola-Malpighi Hospital, Alma Mater Studiorum, University of Bologna, Bologna, Italy; ^4^ Division of Pediatric Surgery Unit, IRCCS Azienda Ospedaliero-Universitaria di Bologna, Bologna, Italy

**Keywords:** MIS-C, COVID-19, inflammation, histology, appendicitis

## Abstract

**Background:**

Multisystem inflammatory syndrome in children (MIS-C) is a rare but serious condition that can potentially develop after SARS-CoV-2 infection in children. Gastrointestinal manifestation in MIS-C can mimic acute abdomen, potentially leading to unnecessary surgical treatment. Immune-mediated mechanisms seem to be a determining factor in its pathogenesis, and histological studies can help to shed light on this aspect. We describe three cases of children diagnosed with MIS-C that underwent appendectomy.

**Methods:**

We retrospectively collected the clinical features and histological findings of three previously healthy children who underwent appendectomy for clinical suspicion of acute appendicitis but were later diagnosed with MIS-C.

**Findings:**

The three children presented with prominent abdominal manifestations and fever leading to the suspicion of acute abdomen. Histological findings showed transmural and perivascular inflammation. Notably, CD68^+^ macrophages were predominant in the child with milder abdominal symptoms without cardiac injury, while CD3^+^ lymphocytes in the patient presented with more severe abdominal pain and cardiovascular involvement at admission.

**Interpretation:**

Gastrointestinal symptoms of children with MIS-C improve after proper immunomodulatory therapy, conversely showing inadequate response to surgical appendectomy. Histological findings revealed different inflammatory cell infiltration that primarily involved perivisceral fat and vessels, and subsequently mucosal tissue, in contrast to other forms of acute appendicitis. Our findings suggest that this kind of peri-appendicitis in MIS-C could represent a focal sign of systemic inflammation, with different histological patterns compared to other forms of acute appendicitis.

## Introduction

Severe acute respiratory syndrome coronavirus 2 (SARS-CoV-2) infection in the pediatric population has a wide clinical spectrum, but it is frequently a mild disease and children usually recover within a few weeks (1).

Respiratory and gastrointestinal (GI) symptoms, such as abdominal pain, vomiting, and diarrhea are common findings in both adults and children during the acute stage of the SARS-CoV-2 infection (2–5).

Following the spread of the Coronavirus Disease (COVID)-19 pandemic, since May 2020, an increasing number of cases of a new condition sharing similarities with Kawasaki disease have been described. The World Health Organization named this newly identified condition as COVID-19-associated multisystem inflammatory syndrome in children (MIS-C) (6). MIS-C is a rare but serious systemic vasculitis, potentially developing four-to-six weeks after SARS-CoV-2 infection in children, characterized by clinical and laboratoristic signs of systemic inflammation and the involvement of two or more organs (7, 8), sometimes requiring intensive care unit (ICU) admission (8, 9). Since abdominal manifestations can be prominent and severe and may mimic acute abdomen, MIS-C can be misdiagnosed as a surgical emergency, potentially leading to unnecessary surgical treatment (8–12). A recent review of patients with MIS-C and severe GI symptoms showed that appendicitis was initially suspected in 5%–30% of patients (13).

The ileum and colon are the most frequently affected sites of the GI tract (2) in MIS-C and common echographic findings are ileitis, colitis, and lymphoadenitis (13), inflammation of the mesenteric adipose tissue, thickening of the terminal ileum and free abdominal effusion (14). Furthermore, MIS-C patients with prominent GI involvement usually exhibit lymphopenia, hypoalbuminemia, and increased D-dimer and fibrinogen levels with remarkably high C-reactive protein (CRP) at blood tests (2, 15). With these features, GI involvement in MIS-C can mimic other inflammatory bowel diseases (2), acute abdomen, and surgical emergencies (16–18) as well as other clinical conditions that can simulate acute appendicitis and thus be classified as pseudo-appendicitis (19).

The pathogenesis of the GI injury in MIS-C is far from being fully understood, although it seems to be mostly ascribable to immune-mediated mechanisms (14). Histological studies can help to shed light on this aspect, but to date, few cases describing the histological alterations of GI involvement in children with MIS-C have been reported in the literature (1, 14, 15). Indeed, when exploratory abdominal surgery was performed in such cases, histological studies revealed diffuse inflammation of the intestine and/or mesenteric lymphadenitis (14), without evidence of a viral cytopathic effect and without detectable viral particles (15).

In an attempt to partially fill this gap, we describe the clinical and histological features of three children affected by MIS-C with prominent abdominal involvement who underwent appendectomy before MIS-C diagnosis was made.

## Materials and methods

We collected the surgical specimens of the appendices from children who underwent a surgical appendectomy in the suspicion of appendicitis and were later diagnosed with MIS-C from March to December 2021.

The diagnosis of MIS-C was done according to the definition of the World Health Organization (6), i.e., multisystemic involvement (at least two of the following symptoms: rash or bilateral non-purulent conjunctivitis or muco-cutaneous inflammation signs, hypotension or shock, features of myocardial dysfunction, pericarditis, valvulitis, or coronary abnormalities, evidence of coagulopathy, acute GI symptoms), in the presence of elevated markers of inflammation (erythrocyte sedimentation rate, (ESR), C-reactive protein, (CRP), or procalcitonin) and fever that affected children and adolescents (0–19 years of age) exposed to SARS-CoV-2 (evidence of COVID-19 as demonstrated by positive RT-PCR test, antigen test or serology, or likely contact with patients with COVID-19) in the previous 2–6 weeks, without other obvious microbial cause of inflammation.

All GI manifestations (vomiting, diarrhea, abdominal pain, abdominal distension, paralytic ileus, pancreatitis, and pseudo-obstruction) were recorded. The presence of vomiting and diarrhea was documented based on standard definition if reported by caregivers and/or directly observed during the acute phase of the hospital stay. Abdominal pain was defined on children's self-reports of pain intensity using a Numerical Rating Scale (NRS) from 0 to 10 (14). Particularly, mild, moderate, and severe pain was defined by a score of at least 4, 6, or 8 out of 10, respectively. GI manifestations were suspected based on clinical presentation, imaging, and laboratory findings.

Blood tests at admission were collected and included: complete blood cell count (white blood cells, WBC; red blood cell count, RBC), CRP, procalcitonin, ferritin, interleukin-6 (IL-6), fibrinogen, D-dimer, alanine-aminotransferase (ALT), aspartate-aminotransferase (AST), albumin, sodium, creatine kinase (CK). Myocardial injury was evaluated by Troponin-I (cTnI) and B-type natriuretic peptide (BNP). In addition, a transthoracic echocardiogram (TTE) was performed in all patients during the acute and subacute phases and at a 6-month follow-up evaluation, to study left ventricular dimensions and function, presence of mitral regurgitation, and coronary dimensions, either as an absolute value (mm) and indexed by z-score.

The treatment of MIS-C consisted of intravenous immunoglobulins (IVIG) at 2 g/kg in a single infusion, methylprednisolone (MPD) at 2 mg/kg/day in case of shock and/or organ-threatening disease, aspirin at 3–5 mg/kg/day and enoxaparin sodium at 100 U/kg twice a day in case of elevation of D-Dimer more than 5 times above the normal values.

Surgical specimens of the appendix were sent to the Pathology Unit and routinely sampled. Two-µm-thick sections were cut from the formalin-fixed paraffin-embedded (FFPE) tissue blocks, for hematoxylin and eosin (H&E) stain and immunohistochemistry (IHC). IHC for CD68 (clone PG-M1) and CD3 (clone 2GV6) was automatically performed by means of automated immunostainer Benchmark® ultra (Ventana Medical Systems, Inc., Roche group, Tucson, AZ, United States), following the manufacturer's instruction. Negative controls are automatically performed at every IHC run by omitting the primary antibody.

Parental informed consent was obtained for all the children (protocol numbers: 98/2016/O/Sper and 178/2021/Sper/AOUBo).

## Results

We collected data from 3 previously healthy Caucasian children (2 boys, aged 7–9 years), presenting to the emergency department (ED) with GI and systemic symptoms starting less than 5 days (median 3 days) before hospitalization. None of them was vaccinated against SARS-CoV-2. One patient (patient 1) presented with hypovolemic shock and one (patient 2) required admission to the ICU because of cardiac dysfunction occurring after the surgical procedure. Clinical and radiological features were all consistent with acute abdomen.

Laboratory tests, echocardiographic alterations, and clinical course of the patients are displayed in Table 1. All patients underwent surgical appendectomy before starting the proper MIS-C treatment.

**Table 1:** Laboratory tests, echocardiographic, and clinical features.

	Patient 1	Patient 2	Patient 3
**Laboratory tests (normal values)**			
WBC (×10^9^/L) (4.8–12)	7.11	6.45	7
Neutrophils (%)	93.2	75.9	68
Lymphocytes (%)	5.1	18.4	16.4
Eosinophils (%)	0.10	0	0
MCV (fl) (76–91)	77	76	58
MCH (pg) (25–31.5)	26.5	26.4	18.8
Fibrinogen (mg/dl) (150–400)	403	445	–
D-dimer test (mg/L) (<0.55)	2.39	1.48	4.08
Creatinine (mg/dl)	0.32	0.30	0.41
Urea (mg/dl) (11–38)	15	21	25
Serum Sodium (mmol/L) (136–145)	130	134	133
Serum Potassium (mmol/L) (3.5–5.3)	4.5	4.2	4.1
Serum Albumin (g/L) (35–50)	31	26.3	38.7
Serum Total Protein (g/dl) (5.7–8)	7.7	7.5	6.2
Tpn I (ng/L) (<11.6)	9	50.9	2.4
BNP (pg/ml) (<100)	–	375	12
Creatine Kinase (UI/L) (<145)	42	31	45
IL-6 (pg/ml) (<6.4)	27.4	5.1	7.7
Ferritin (pg/ml) (11–306)	394	–	163
CRP (mg/dl) (<0.5)	16.81	19.25	18.66
Procalcitonin (ng/ml) (<0.5)	17.50	20.8	3.8
**Echocardiography at admission**			
Mitral regurgitation (yes/no)	No	Yes	No
Pericardial effusion (yes/no)	No	No	No
LVEF (%)	52	47	59
**Clinical Data**			
ICU admission (yes/no)	No	Yes	No
LOS (days)	10	8	7
Total days of fever	7	3	3
Time from the onset of fever to proper MIS-C treatment (days)	5	3	4

Legend: WBC, white blood cells; N, neutrophiles, L, lymphocytes, RBC, red blood cells; Hb, hemoglobin; PLT, platelets count; AST, aspartate-aminotransferase; ALT, alanine-aminotransferase; Tpn, Troponin I; BNP, brain natriuretic peptide; IL-6, Interleukin-6; CRP, C reactive protein; LVEF, left ventricular ejection fraction; ICU, intensive care unit; LOS, length of hospital stay.

The median hospital stay was 8 days.

**Patient 1:** A 7-year-old boy presented to the ED with a four-day history of vomiting, diarrhea, abdomen distension, and severe pain to the lower right abdomen (8/10 on NRS). Upon admission, he was pale and drowsy, febrile (40.5°C), tachycardic (135 bpm), and hypotensive (80/40 mmHg). His abdomen was diffusely tense and distended with a positive Blumberg sign. Two 10 ml/kg-boluses of isotonic crystalloid solution were given with subsequent improvement of vital signs. Blood tests revealed increased inflammatory markers, anemia, and thrombocytopenia. Abdominal ultrasound showed marked terminal ileitis with inflammation extending through the ascending colon, mesenteric fat stranding, and mesenteric lymphadenopathy in the right lower quadrant; the appendicular diameter was 6 mm. Echocardiography documented a mild decrease in left ventricular ejection function (LVEF) and dilation of the right coronary artery (*z*-score 2.3).

**Figure 1:**
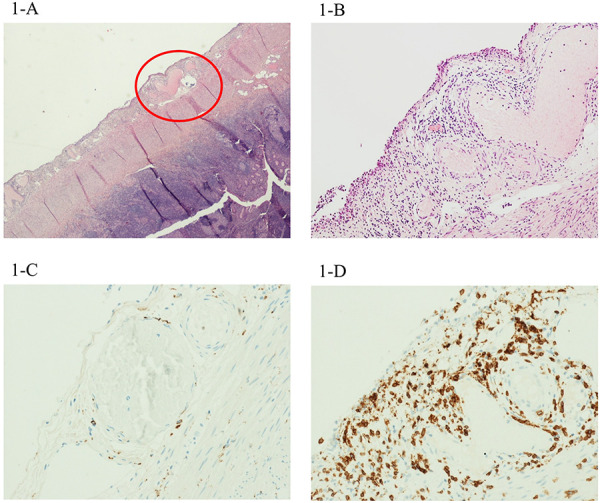
H&E image of the first case, with moderate-to-severe periappendiceal inflammation, involving the wall of perivisceral veins. This infiltrate showed relatively few CD68^+^ macrophages (**C**) and several CD3^+^ lymphocytes (**D**). Magnification 4× (**B**) and 20× (**A**).

In the suspect of acute appendicitis, he underwent transumbilical laparoscopic-assisted appendectomy (TULAA). Endoscopic evaluation of the appendix did not show frank inflammatory signs. The main histopathological features revealed moderate-to-severe peri-appendiceal inflammation and neoangiogenesis. Particularly, the visceral wall and perivisceral veins were infiltrated predominantly by CD3^+^ lymphocytes, with a minor amount of CD68^+^ macrophages (Figure 1).

After surgery, his clinical condition did not improve: he was prostrated and presented with high-grade fever, and diffuse abdominal pain persisted. Familiar history turned out to be positive for COVID-19 five weeks before, so the child was tested for SARS-CoV-2 serology, showing positive for previous infection. A diagnosis of MIS-C was thus made and the treatment with IVIG, MPD, acetylsalicylic acid, and enoxaparin sodium was started. After 48 h his clinical status and abdominal manifestations improved. At the 6-month follow-up, cardiac function and coronary dimensions had returned to normal.

**Patient 2:** A 9-year-old boy presented to the ED with a four-day course of fever (39.5°C) and asthenia, nausea, and moderate abdominal pain (6/10 on NRS). He presented with a tense and tender abdomen, particularly in the right lower quadrant, and positive Blumberg sign; moreover, he had signs of mild dehydration (dry mucous membranes and skin, fuzzy tongue, dull eyes), but his vital signs were normal (blood pressure 108/70 mmHg, heart rate 110 bpm, refill time <2 s, blood oxygen saturation levels 100% in room air). Blood tests showed increased markers of inflammation, anemia, thrombocytopenia, and slightly elevated cTnI. Abdominal ultrasound showed aperistaltic and dilated appendix (11 mm) with echogenic prominent perivisceral fat.

**Figure 2:**
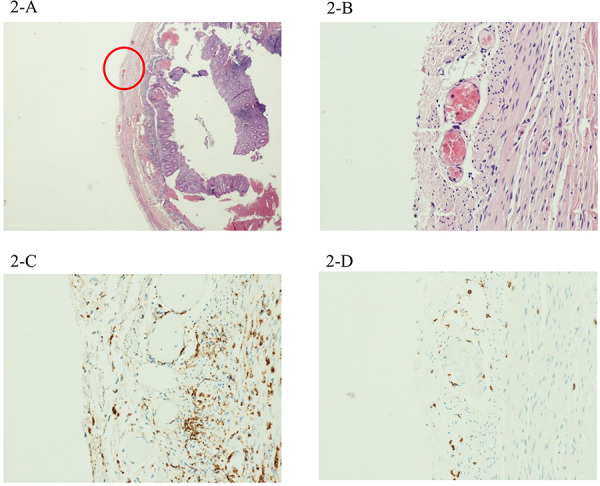
The second case showed mild inflammation, but with a perivascular involvement mostly by CD68^+^ macrophages (**C**), with very few CD3^+^ lymphocytes (**D**). Magnification 4× (**B**) and 20× (**A**).

Intravenous rehydration with an isotonic solution was started and video-laparoscopic appendectomy was performed on the suspect of acute appendicitis. Histopathological findings showed mild transmural and perivascular inflammation. The inflammatory infiltrates were dominated by CD68^+^ macrophages and CD3^+^ lymphocytes in equal distribution (Figure 2).

After 48 h, his clinical conditions worsened with persistent high-grade fever, oxygen desaturation (85% in room air), and systemic shock (78/34 mmHg). Echocardiography documented mild left ventricular dysfunction (EF: 47%) and mitral insufficiency, while coronary arteries diameters were normal. He was, therefore, admitted to ICU requiring intensive monitoring and inotropic and respiratory support. Chest x-ray showed signs of pulmonary edema with bilateral pleural effusion and basal congestion. In the suspicion of an MIS-C, SARS-CoV-2 serology was performed, resulting positive for previous infection, despite negative familiar and personal history of COVID-19 and no vaccination. The proper therapy for MIS-C was started with subsequent improvement after 36 h.

He gradually recovered and was discharged after 8 days of hospitalization. At the 6-month follow-up, cardiac function and mitral regurgitation had returned to normal.

**Patient 3:** An 8-year-old girl was admitted to the ED for a high-grade fever (39.5 °C) lasting for 3 days, headache, and lower limbs muscular pain. Personal history was positive for an asymptomatic SARS-CoV-2 infection three weeks before the onset of abdominal pain. At admission, she was suffering from mild right lower abdominal pain and diffuse abdominal distension with positive Blumberg sign (5/10 on NRS), vomiting, and diarrhea; vital signs were normal (blood pressure 108/71 mmHg, heart rate 125 bpm, blood oxygen saturation 99% in room air). Abdominal ultrasound showed a mild thickening of the ileal wall to ascending colon and perivisceral fluid in the Douglas' pouch, without clear signs of acute appendicitis. Cardiac function and coronary arteries were normal.

**Figure 3:**
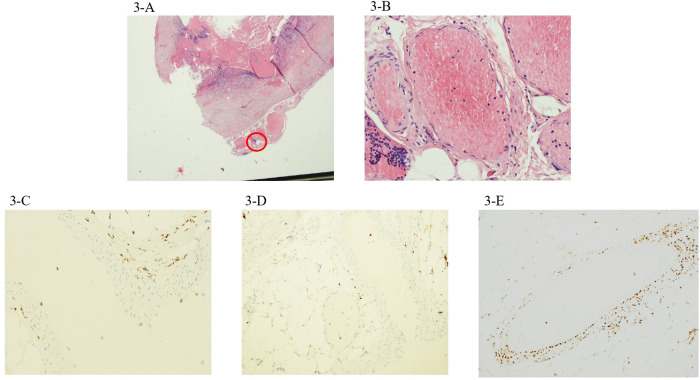
The third case showed mild inflammation, with a scarcity of both perivascular CD68^+^ macrophages (**C**) and CD3^+^ lymphocytes (**D**). Magnification 4× (**B**) and 20× (**A**). A detail of the CD68^+^ macrophagic infiltrate around and within a large periappendiceal vein in case 2 (**E**).

She underwent to explorative laparoscopy procedure with complementary laparoscopic appendectomy. Histological findings revealed mild transmural and perivascular inflammation with an inflammatory infiltrate mostly represented by CD68^+^ macrophages with very few CD3^+^ lymphocytes (Figure 3).

In the suspicion of MIS-C, she started standard treatment with prompt improvement. She was discharged after 7 days.

## Discussion

Children affected by MIS-C can experiment moderate-to-severe GI symptoms that appear to be associated with increased inflammatory markers, a more severe clinical course, and an increased risk of admission to the ICU (2). Furthermore, GI involvement in MIS-C can mimic acute abdomen, requiring differential diagnosis with surgical emergencies, particularly appendicitis, eventually leading to exploratory laparotomies (14). Nevertheless, laparoscopic or surgical appendectomy in children with MIS-C is not usually indicated, since it does not seem to improve the clinical course of the disease, and exposes these already critically ill patients to unnecessary anesthesiologic and surgical risks (13).

The histopathologic features in MIS-C described so far showed transmural and vascular inflammation of the affected GI tract. Particularly, transmural lymphocytic inflammation and focal acute enteritis involving mesentery and adjacent vessels of the ileum without appendicular involvement was described in a patient undergoing ileocolic resection: venous microthrombi originated in the subendothelial space widely affected mucosa, submucosa, and subserosa of the terminal ileum (15).

In our patients, the main histological findings were inflammatory infiltrates involving the wall of several arteries and veins in the peri-appendiceal fat, with associated neoangiogenesis. IHC revealed that the composition of this perivascular transmural infiltrate significantly differed among patients according to clinical severity. Particularly, perivascular inflammation was characterized by a significant proportion of CD68^+^ macrophages in the child with milder abdominal symptoms without cardiac injury (case 3), while CD3^+^ lymphocytes were predominant in the patient presenting with more severe abdominal pain and cardiovascular involvement at admission with shock, ventricular dysfunction, and coronary dilation.

Notably, in the usual presentation of acute appendicitis, a predominantly neutrophilic granulocyte-rich inflammation typically starts in the mucosa, eventually spreading through the wall up to the serosa. Indeed, perivisceral inflammation is described in more than half of cases of acute pediatric appendicitis, mostly with moderate-to-severe inflammation (20). In our cases, inflammation of the serosa was present even if mucosal inflammation was none-to-mild, in line with previous observations on the extra-pulmonary manifestations of SARS-CoV-2 disease (21, 22).

It is difficult to assess whether this acute peri-appendicitis is just a comorbidity in children with MIS-C or if it is a clinical and histopathological part of the systemic inflammatory process, potentially involving multiple organs and systems. Albeit local, perivascular inflammation in the appendix of children with MIS-C likely represents the focal sign of a diffuse disease and a systemic inflammation involving the entire GI tract and potentially other sites. Strong support for the systemic nature of MIS-C to intestinal and appendicular inflammation comes from the response to proper MIS-C treatment, since all patients rapidly improved after immunomodulatory therapy, rather than after surgical procedure. It is still unclear whether the inflammatory findings are determined by direct viral-induced cellular damage or if they are the consequence of a systemic inflammatory process affecting the GI tract (15, 23).

Of note, neither viral particles nor viral cytopathic effect was detected in any of the histopathological reports published so far (15, 21), highly suggesting an immune-mediated mechanism rather than a direct viral injury. Furthermore, the pathological findings from the three cases show that inflammatory infiltrate primarily affects the perivisceral fat and vessels, in contrast with other forms of acute appendicitis in which inflammation extends from the mucosal surface to the perivisceral fat.

Interestingly, “pseudo-appendicitis” can be the signature of other diseases, such as inflammatory bowel diseases (IBD), mainly Crohn's disease (CD), *Bartonella*-related ileitis, *Yersinia enterocolitica* infection, and Kawasaki disease (KD) with intestinal involvement, with whom differential diagnosis must be considered (24). *Bartonella henselae*, for instance, causes infectious diseases that can present as acute ileitis and systemic symptoms such as fever and lymphoadenitis (25, 26). *Y. enterocolitica* (27) and *Yersinia pseudo-tuberculosis* (19) infections share similar clinical features, blood test alterations, and abdominal echographic findings with acute appendicitis. Histological findings in *Y. enterocolitica* infection are aspecific, documenting a peri-appendicitis characterized by mixed acute and chronic inflammation infiltrates, focal neutrophilic cryptis, and epithelial cell granulomas composed by small T-lymphocytes, plasma monocytes, and hystiocytes (28). However, diagnosis of *Y. enterocolitica* infection is made by isolation of the germ from biological samples (27).

On the other hand, non-infectious systemic diseases, such as IBD and KD can also mimic acute appendicitis, with peculiar histological features that differ from MIS-C pathological findings.

When the appendix is concerned, transmural inflammation with fibrous thickening of the wall, non-caseating granulomas, muscular hypertrophy, and crypt abscesses are documented (29). Typical extra-intestinal manifestations such as arthralgia, uveitis, and arthritis (30), and Anti-*Saccharomyces cerevisiae* antibodies can lead to a diagnosis of CD.

Appendicitis in KD is very rare, as opposed to frequent abdominal symptoms (31). A large number of IgA plasma cells, likely due to the stimulation of the systemic IgA immune system, were found in the GI tract in patients with KD, but also in non-KD controls (32). In the 8 cases of KD with appendicitis published so far, histological findings showed found focal inflammation with neutrophilic and eosinophilic infiltration in one patient and transmural inflammation and arteritis in another one (33). In case of persistent postoperative fever, especially if other systemic signs appear, the diagnosis should be reconsidered, with the awareness that KD tends to affect younger patients than MIS-C (34).

## Conclusion

Our findings support the hypothesis that peri-appendicitis in children with MIS-C might represent a manifestation of multisystemic inflammation. Notably, the perivascular transmural infiltrate differed among patients with a significant proportion of CD3^+^ lymphocytes in presence of more severe abdominal pain and cardiovascular involvement at admission, while CD68^+^ macrophages were predominant when intestinal symptoms were milder without cardiac injury. The cell infiltration patterns could suggest that different cells are involved in the inflammatory response and may indicate different stages or expressions of the same pathological process. Pediatricians and pediatric surgeons must be aware of this clinical presentation of MIS-C in order to properly treat the patients and avoid unnecessary surgery.

## Data Availability

The raw data supporting the conclusions of this article will be made available by the authors, without undue reservation.
